# Effects on Rotational Dynamics of Azo and Hydrazodicarboxamide-Based Rotaxanes †

**DOI:** 10.3390/molecules22071078

**Published:** 2017-06-28

**Authors:** Adrian Saura-Sanmartin, Juan S. Martinez-Espin, Alberto Martinez-Cuezva, Mateo Alajarin, Jose Berna

**Affiliations:** Departamento de Química Orgánica, Facultad de Química, Regional Campus of International Excellence “Campus Mare Nostrum”, Universidad de Murcia, 30100 Murcia, Spain; adrian.saura@um.es (A.S.-S.); j.s.m.espin@smn.uio.no (J.S.M.-E.); alajarin@um.es (M.A.)

**Keywords:** template synthesis, hydrogen bonded rotaxanes, pirouetting motion, NMR studies

## Abstract

The synthesis of novel hydrogen-bonded [2]rotaxanes having two pyridine rings in the macrocycle and azo- and hydrazodicarboxamide-based templates decorated with four cyclohexyl groups is described. The different affinity of the binding sites for the benzylic amide macrocycle and the formation of programmed non-covalent interactions between the interlocked components have an important effect on the dynamic behavior of these compounds. Having this in mind, the chemical interconversion between the azo and hydrazo forms of the [2]rotaxane was investigated to provide a chemically-driven interlocked system enable to switch its circumrotation rate as a function of the oxidation level of the binding site. Different structural modifications were carried out to further functionalize the nitrogen of the pyridine rings, including oxidation, alkylation or protonation reactions, affording interlocked azo-derivatives whose rotation dynamics were also analyzed.

## 1. Introduction

**Figure 1:**
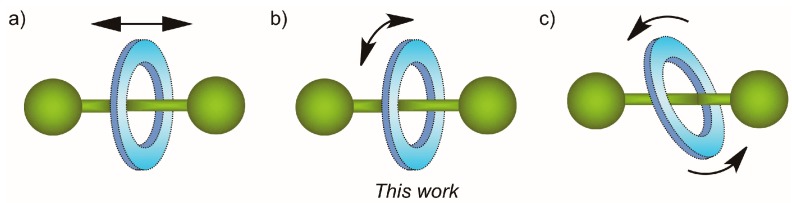
Schematic representation of the most common molecular motions in [2]rotaxanes: (**a**) translation; (**b**) rotation or *pirouetting*; and (**c**) *rocking*.

Mechanically interlocked compounds are chemical entities composed by, at least two or more entwined components [1,2,3]. The lacking of a covalent bond between the subunits of these compounds allows relative large amplitude motions, such as translation, rotation (*pirouetting*) or *rocking* (Figure 1) [4,5]. During the last decades, the control of the internal dynamics of these species under different external stimuli allowed the development of smart interlocked devices for a broad range of applications [6,7,8,9,10,11,12].

The synthesis of hydrogen-bonded rotaxanes requires the presence of suitable binding sites able to template the formation of the corresponding tetralactam macrocycle. In this sense, the employment of 1,4-dicarboxamides [13,14,15], nitrones [16,17], squaraines [18,19] or di(acylamino)pyrididines [20,21,22,23], together with hydrogen-bonding templation with anions [24,25] can be highlighted. In this arena, some of us reported that azodicarboxamides are able to act as effective templates for driving the assembly of hydrogen-bond-assembled [2]rotaxanes [26,27,28]. Moreover, these binding sites can be efficiently interconverted with their reduced hydrazo forms through hydrogenation-dehydrogenation of its nitrogen-nitrogen bond. This type of chemical control was efficiently employed for building stimuli-responsive molecular shuttles [26]. In a different work, the rotation dynamics of these particular systems were shown to depend on the oxidation level of the nitrogen-based binding site [27].

In polyamide-based rotaxanes, the thread-macrocycle affinity can be modulated by the variation of the substituents on the macrocyclic counterpart. Indeed, the presence of electron-donor or electron-withdrawing groups can strengthen or weaken this interaction, breaking or accelerating the rotational motion of the ring [29,30]. In pyridine-containing macrocycles a chemical variation of the pyridyl nitrogen can also undergo changes on the rotational speed [31].

Herein we describe the effect of integrating dicyclohexylmethyl stoppers on an azodicarboxamide template in the preparation of a hydrogen-bond-assembled [2]rotaxane having two pyridine rings into the macrocycle and its interconversion into its corresponding hydrazo surrogate. In addition, some chemical modifications of the pyridine moiety were also achieved, including protonation, oxidation and alkylation reactions for providing a novel set of azodicarboxamide-based rotaxanes. Finally the rotational dynamics of each interlocked molecule was studied by temperature-dependent NMR experiments.

## 2. Results

### 2.1. Synthesis of Threads [2H]-***2*** and ***2***

**Scheme 1:**
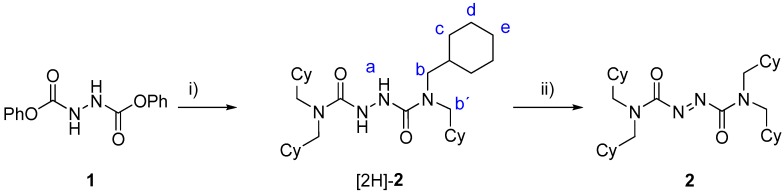
Synthesis of the hydrazodicarboxamide [2H]-**2** and its azo derivative **2**. *Reagents and conditions:* (**i**) (CyCH_2_)_2_NH, Et_3_N, CHCl_3_, reflux, 24 h, 51%; (**ii**) NBS (*N*-bromosuccinimide), pyridine, CH_2_Cl_2_, 25 °C, 1 h, 97%. (Cy = cyclohexyl, C_6_H_11_).

The reaction of diphenyl hydrazodicarboxylate (**1**) with bis(cyclohexylmethyl) amine in the presence of two equivalents of triethylamine provided the hydrazodicarboxamide [2H]-**2** in a 51% yield. Next, the oxidation of the hydrazo compound [2H]-**2** was carried out using *N*-bromosuccinimide in the presence of pyridine. This reaction was monitored by IR spectroscopy observing the vanishing of the band ascribed to the NH at 3397 cm^−1^ of the hydrazo compound and the shifting of the absorption frequency of the CO band from 1653 cm^−1^ to 1699 cm^−1^. After the work-up, the azodicarboxamide **2** was isolated in a 97% yield (Scheme 1).

### 2.2. Synthesis of Rotaxanes [2H]-***3*** and ***3***

**Scheme 2:**
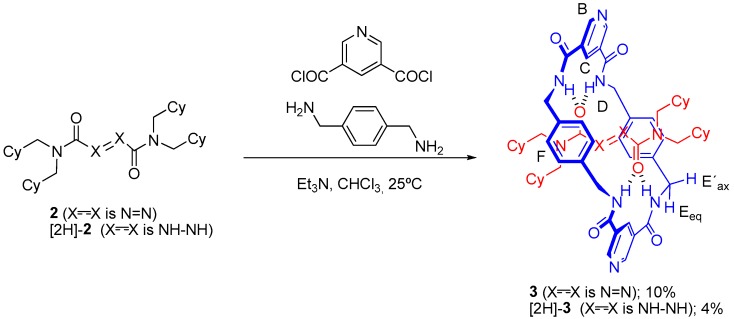
Syntheses of the azo and hydrazo rotaxanes **3** and [2H]-**3**. *Reagents and conditions:* *p*-xylylenediamine, 3,5-pyridinedicarbonyl dichloride, Et_3_N, CHCl_3_, 25 °C, 4 h.

The five-component clipping reaction between *p*-xylylenediamine, 3,5-pyridinedicarbonyl dichloride, the hydrazo template [2H]-**2** and triethylamine provided the hydrazo[2]rotaxane [2H]-**3** in only 4% yield (Scheme 2). The rotaxane formation reaction using the template **2** leads to the interlocked azo compound **3** in a more reasonable 10% yield thus probing the better templating capability of the azo compound for driving the construction of the tetralactam ring around the thread (Scheme 2).

These results are in line with those obtained for the assembly of *N*,*N*,*N′*,*N′*-tetrabenzyl-hydrazodicarboxamide and its corresponding azo derivative [26]. The hydrazodicarboxamide threads likely adopt a ground state conformation in which the carbonyl hydrogen bond acceptors do not have the ideal orientation of an efficient template [32]. However, the incorporation of two pyridine rings into the tetralactam ring also provokes a noticeable decrease of the yield probably due to a non-ideal aggregation between the thread and the intermediate polyamides, precursors of the interlocked tetralactam [29].

### 2.3. Analysis of Threads ***2*** and Rotaxanes ***3***

**Figure 2:**
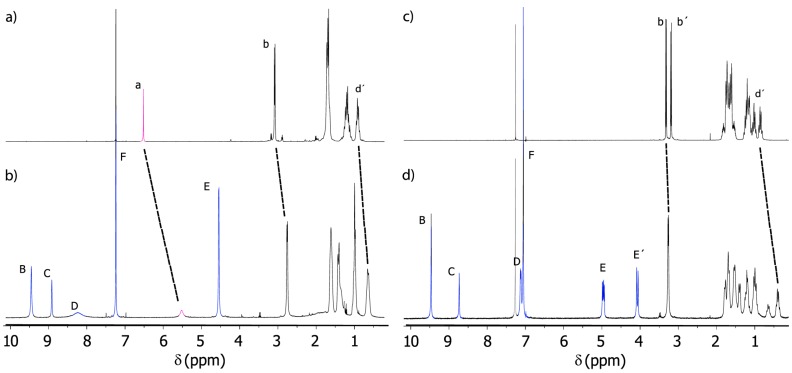
^1^H-NMR spectra (400 MHz, CDCl_3_, 298 K) of (**a**) hydrazodicarboxamide thread [2H]-**2**; (**b**) hydrazodicarboxamide rotaxane [2H]-**3**; (**c**) azodicarboxamide thread **2**; and (**d**) azodicarboxamide rotaxane **3**. See Scheme 1 and Scheme 2 for signal assignments.

Figure 2 displays the stacked plot of the ^1^H-NMR spectrum of each thread [2H]-**2** and **2** and the corresponding interlocked compounds [2H]-**3** and **3**. Both comparisons prove the interlocked nature of the assembled products. Nevertheless, the shape of the signal of the macrocyclic methylenic protons H_E_ of these rotaxanes deserves to be highlighted. Whereas the signals ascribed to the macrocyclic methylene protons of [2H]-**3** (Figure 2b) appears as a slightly broad singlet a 4.56 ppm, the analogue signals of **3** (Figure 2d) are non-equivalent as result of the different magnetic environments attributed to its equatorial (H_E_) and axial (H_E_’) positions in the macrocycle. The peak for the equatorial methylene proton emerges as a doublet of doublets (^2^*J*_(H1,H2)_ = 14.3 Hz and ^3^*J*_(H1,HD)_ = 7.3 Hz) at 4.96 ppm and the peak for the axial one appears as a doublet (*J*_(H1,H2)_ = 14.1 Hz) at 4.06 ppm. This patent difference is connected with the rotational motion of the ring of single binding site amide-based [2]rotaxanes [16,17,29,30,31,33,34,35,36,37] which occurs at a higher spinning rate in [2H]-**3** than in **3**. As usually occurred in polyamide-based rotaxanes, peaks corresponding to the hydrogens of the thread are shifted to lower ppm values due to the presence of the macrocycle around it. Interestingly, the set of signals referred to the hydrogens H_b_ and H_b_’ in thread **2**, which indicates a slow rotation motion of the amide CO-N bond, emerges in only one signal in **3** after rotaxane formation. Moreover, the substantial upfield shift (Δδ ≈ 0.5 ppm) corresponding to the signal of the methylenic H_d_’ protons of one of the cyclohexane rings of **3** (Figure 2d) with respect to the naked thread (Figure 2c) reveals the establishment of stabilizing CH···π interactions [38,39] which further decelerated the rotational motion [29]. In contrast a variation of the chemical shift in about 0.25 ppm was observed when compared thread [2H]-**2** (Figure 2a) and rotaxane [2H]-**3** (Figure 2b) owing to the shielding effect of the aromatic rings of the macrocycle.

### 2.4. Chemical Interconversion between Rotaxanes ***3***

**Scheme 3:**
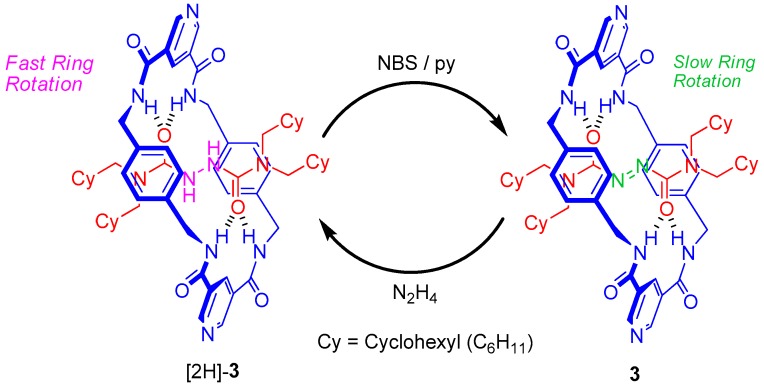
Interconversion of the azo and hydrazo rotaxanes **3** and [2H]-**3**.

Taking into account the above considerations on the spinning rate of the macrocycle of the interlocked systems, most probably we could switch between two different dynamic states just by exchanging the oxidation state of the dinitrogenated binding site of these rotaxanes (for related compounds, see reference [27]). This is why we assayed the chemical interconversion between the two accessible oxidation states of [2]rotaxanes **3**. A reduction of the azo rotaxane **3** with hydrazine quantitatively afforded the [2]rotaxane [2H]-**3** (Scheme 3). In the reverse sense, [2H]-**3** was completely transformed by oxidation with *N*-bromosuccinimide in the presence of pyridine. In this way, we switched from a fast ring rotation state of a rotaxane to a slow ring rotation one, and viceversa, by simple and efficient chemical modifications.

### 2.5. Chemical Functionalization of the Pyridine Rings of the Macrocycle of Rotaxane ***3***

**Scheme 4:**
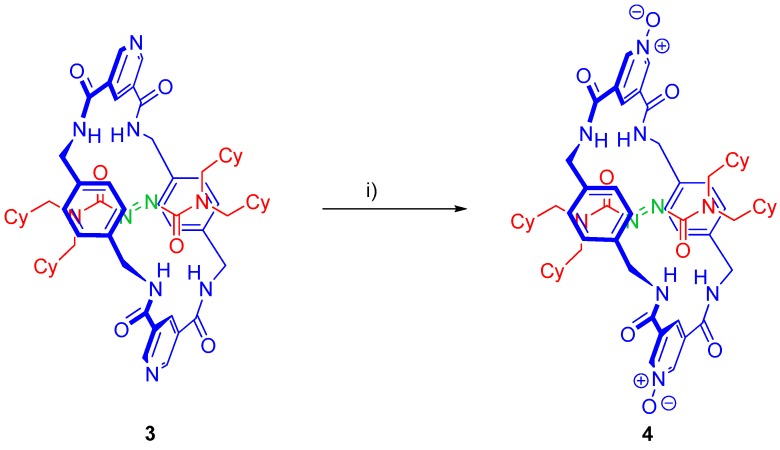
Synthesis of the azodicarboxamide-based rotaxane **4**. *Reagents and conditions: m-*CPBA (3 equiv.), CHCl_3_, 25 °C, 3 h.

We next evaluated the changes on the pirouetting motion of the macrocycle following some modifications at the pyridinic nitrogen atom. We envisaged that this could modify the affinity of the macrocycle for the azo-based binding site and, consequently, its rotational dynamics. Following this idea we synthesized the azo-derived rotaxane **4** from compound **3**, in which the two nitrogen atoms of the pyridine rings were chemically converted into *N*-oxides by reaction with *m*-chloroperbenzoic acid (*m*-CPBA) (Scheme 4). The reaction proceeded smoothly at room temperature obtaining a high yield of the corresponding *N*-oxide derivative **4**.

**Scheme 5:**
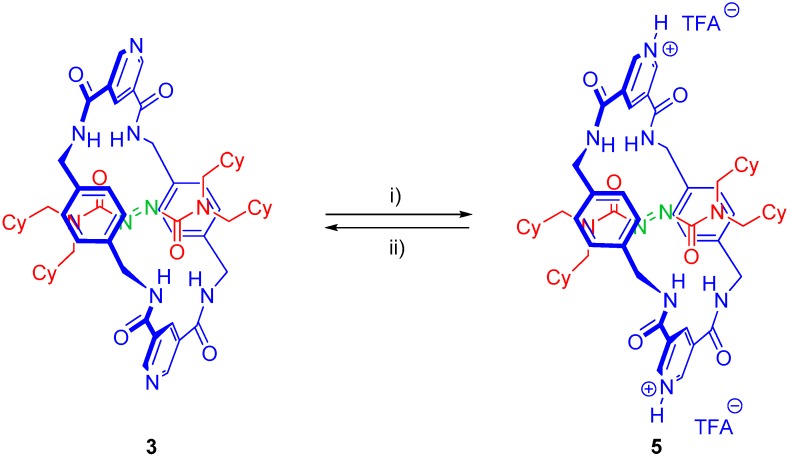
Synthesis of the TFA salt azodicarboxamide-based rotaxane **5**. *Reagents and conditions:* (**i**) trifluoroacetic acid, CH_3_Cl, 25 °C, 1 h; (**ii**) Amberlyst^®^ A21, CH_3_Cl, 25 °C, 1 h.

Due to the inherent basicity of the pyridine moiety, the corresponding pyridinium trifluoacetate salt **5** can be easily prepared by treatment of rotaxane **3** with an excess of trifluoacetic acid (Scheme 5). We expected that the double protonation of the tetralactam macrocycle can induce an acceleration of the pirouetting motion around the thread as happened in other fumaramide-based rotaxanes [31]. The corresponding salt **5** was obtained in quantitative yield after complete evaporation of the excess of trifluoroacetic acid under high vacuum. Additionally, the initial compound **3** was easily recovered by deprotonation with the weakly basic resin Amberlyst^®^ A21.

**Scheme 6:**
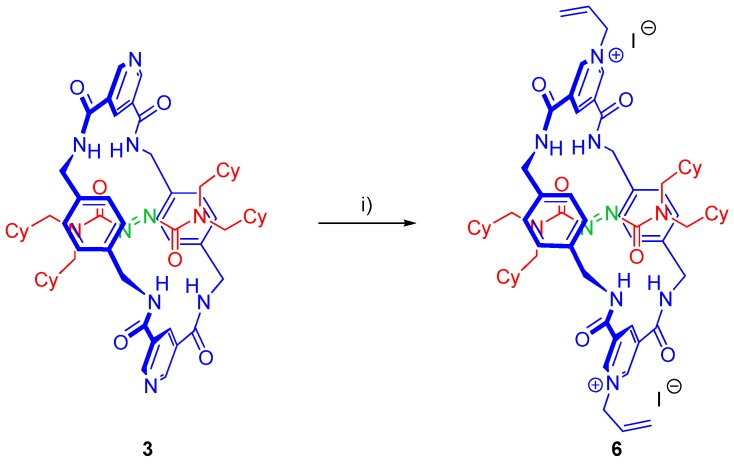
Synthesis of the azodicarboxamide-based rotaxane **6**. *Reagents and conditions:* (**i**) allyl iodide (3 equiv.), CH_3_CN, 80 °C, 48 h.

Another modification that can be achieved over the pyridine moiety is the *N*-alkylation with different aliphatic electrophiles, such as alkyl halides. Indeed, the reaction between rotaxane **3** and allyl iodide provided the corresponding rotaxane **6** in high yield, which was insoluble in chlorinated solvents (Scheme 6). A counteranion exchanged (SbF_6_^−^ and PF_6_^−^) was tested to improve the solubility of the system, but these attempts were fruitless.

### 2.6. Variable-Temperature ^1^H-NMR Experiments: Calculation of the Rotational Barrier

The estimation of the ring affinity towards the binding sites in interlocked molecular shuttles is crucial for a correct control of their internal translational motion. In benzylic amide [2]rotaxanes, the energy barrier for the rotation of the macrocycle around the template is directly correlated to their affinity [16,29,30,31,33,34,35]. Having this in mind, we decided to calculate the energy barrier of rotation of the pyridine-based tetralactam ring in the synthesized rotaxanes **3** and [2H]-**3**, and in their corresponding derivatives **4**–**5** by variable-temperature (VT) NMR experiments (see Supporting Information). Note that if the interactions between the macrocycle and thread are weak enough a fast exchange between the methylene protons (H_E_) of the macrocycle should be observed. The rate of this process would decrease at lower temperatures, resulting in the splitting of the singlet corresponding to the hydrogens located in axial (H_Eax_) and equatorial (H_E’eq_) positions in the ^1^H-NMR spectrum.

**Figure 3:**
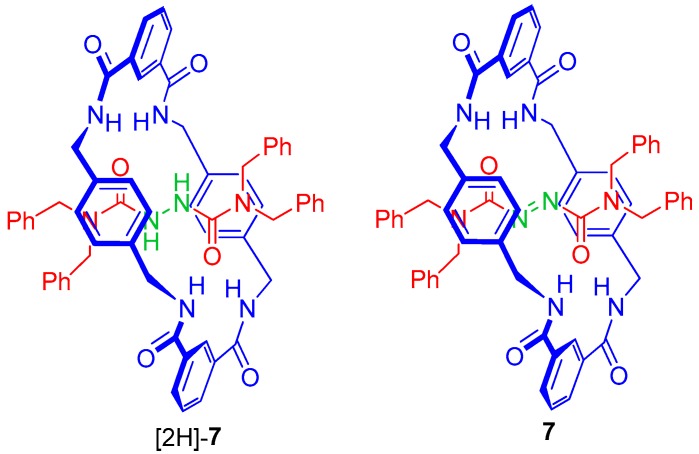
Tetrabenzyl-substituted hydrazo- and azo-derived rotaxanes [2H]-**7** and **7** [27].

The rate of rotation of [2H]-**3** is higher than the one of the azo-derivative **3**, indicating a lower level of interaction between the reduced thread and the macrocycle, and thus a faster ring rotation (Table 1, entries 1–2). This is fully consistent with the reported data in related azo- and hydrazo-based rotaxanes [26,28]. Thus, the energy barrier in [2H]-**3** (10.7 kcal·mol^−1^) is very similar to the calculated one in the previously reported rotaxane [2H]-**7** (10.4 kcal·mol^−1^), bearing four tetrabenzyl groups as stoppers and the classic macrocyclic component (Figure 3) (Table 1, entries 1 and 5). On the other hand, when comparing the energy barrier values of rotaxanes **3** (19.1 kcal·mol^−1^) and **7** (14.8 kcal·mol^−1^) a huge effect on the dynamics is observed due mainly to the presence of different stoppers (Table 1, entries 2 and 6). The higher energy barrier in rotaxane **3** can be explained by the establishment of cooperative CH···π interactions between the macrocycle and the cyclohexyl stoppers [29], in combination with the increase of the steric hindrance of the non-planar cyclohexyl groups compared with the planar phenyl rings, which slow down the spinning rate of the macrocycle. It should be note that dissimilar ring affinity of the azo and hydrazo binding sites in the rotaxane **3** and [2H]-**3** (8.4 kcal·mol^−1^) is one of the highest energy gaps reported for switchable binding sites. The oxidation of the pyridine rings to the corresponding *N*-oxide makes the macrocycle in compound **4** to rotate slightly faster when compared with the ring of the starting azo-based rotaxane **3** (Table 1, entries 2–3). Whereas the macrocycle in the trifluoroacetate salt **5** rotates at nearly the same rate than in the neutral compound, in contrast with the reported data in related hydrogen-bonded rotaxanes [31]. Unfortunately, in the case of the allyl-dipyridinium derivative **6** we were not able to perform VT NMR experiments due to its poor solubility in chlorinated solvents (CDCl_3_ or C_2_D_2_Cl_4_).

## 3. Materials and Methods

### 3.1. General Information

Unless stated otherwise, all reagents were purchased from Aldrich Chemicals (St. Louis, MO, USA) and used without further purification. HPLC grade solvents (Scharlab, Barcelona, Spain) were nitrogen saturated and were dried and deoxygenated using a Pure-Solv 400 Solvent Purification System (Innovative Technology Inc., Amesbury, MA, USA). Column chromatography was carried out using silica gel (60 Å, 70–200 μm, SDS) as stationary phase, and TLC was performed on precoated silica gel on aluminun cards (0.25 mm thick, with fluorescent indicator 254 nm, Fluka Chemie AG, Buchs, Switzerland) and observed under UV light. All melting points were determined on a Kofler hot-plate melting point apparatus and are uncorrected. ^1^H-, ^19^F- and ^13^C-NMR spectra were recorded at 298 K on Avance 300 and 400 MHz instruments (Bruker, Karlsruhe, Germany). ^1^H-NMR chemical shifts are reported relative to Me_4_Si and were referenced via residual proton resonances of the corresponding deuterated solvent whereas ^13^C-NMR spectra are reported relative to Me_4_Si using the carbon signals of the deuterated solvent. Signals in the ^1^H and ^13^C NMR spectra of the synthesized compounds were assigned with the aid of DEPT, APT, or two-dimensional NMR experiments (COSY, HMQC and HMBC). Abbreviations of coupling patterns are as follows: br, broad; s, singlet; d, doublet; t, triplet; q, quadruplet; m, multiplet. Coupling constants (J) are expressed in Hz. High-resolution mass spectra (HRMS) were obtained using a time-of-flight (TOF) instrument (Agilent 6220, Agilent Technologies Inc., Santa Clara, CA, USA) equipped with electrospray ionization (ESI). IR spectra were registered on a Nicolet Avatar 360 FTIR spectrophotometer (Thermo Electron Corporation, Madison, WI, USA).

### 3.2. Synthesis of Threads

#### 3.2.1. Synthesis of *N*^1^,*N*^1^,*N*^2^,*N*^2^-Tetrakis(cyclohexylmethyl)-1,2-hydrazodicarboxamide ([2H]-**2**)

To a solution of diphenyl hydrazodicarboxylate (**1**) (1.65 g, 6.06 mmol) in chloroform (50 mL) was added bis(cyclohexylmethyl)amine (2.80 g, 13.37 mmol) followed by triethylamine (1.69 mL, 12.16 mmol). The reaction mixture was stirred for 15 hours at reflux temperature after which time the reaction was concentrated under reduced pressure and purified by column chromatography on silica gel using a CHCl_3_/MeOH (99/1) mixture as eluent to give the title product as a white solid ([2H]-**2**, 1.53 g, 51%). m.p. = 249–251 °C. IR (Nujol): 3627 (w), 3575 (w), 3397 (w), 3019 (w), 2925 (w), 1653 (w), 1449 (w), 1215 (vs), 1046 (w), 758 (vs), 699 (s). ^1^H-NMR (400 MHz, CDCl_3_) δ: 6.54 (s, 2H, NH), 3.09 (d, *J* = 6.9 Hz, 8H, NC*H*_2_), 1.82–1.53 (m, 24H, H_Cy_), 1.34–1.05 (m, 12H, H_Cy_), 1.02–0.75 (m, 8H, H_Cy_). ^13^C-NMR (75 MHz, CDCl_3_) δ: 159.2 (CO), 54.5 (CH_2_), 36.9 (CH), 31.1, 26.6, 26.0; HRMS (ESI) calcd. for C_30_H_55_N_4_O_2_ [M + H]^+^ 503.4325, found 503.4329.

#### 3.2.2. Synthesis of *N*^1^,*N*^1^,*N*^2^,*N*^2^-Tetrakis(cyclohexylmethyl)-1,2-azodicarboxamide (**2**)

To a solution of the 1,2-hydrazodicarboxamide [2H]-**2** (2.87 g, 5.71 mmol) in dichloromethane (50 mL) were added pyridine (0.6 mL, 6.85 mmol) and *N*-bromosuccinimide (1.12 g, 6.28 mmol). The resulting orange solution was stirred at room temperature for 1 h. Then the reaction mixture was diluted with dichloromethane (100 mL) and sequentially washed with water (100 mL), 5% aqueous solution of Na_2_S_2_O_3_ (75 mL) and saturated solution of NaHCO_3_ (2 × 75 mL). The organic phase was dried with anhydrous MgSO_4_, and concentrated under reduced pressure to afford a crude material which was purified by column chromatography on silica gel eluting with a CHCl_3_/MeOH (98/2) mixture as eluent to give the title product as an orange solid (**2**, 2.77 g, 97%). m.p. = 187–189 °C. IR (Nujol): 3019 (m), 2928 (w), 1699 (m), 1450 (w), 1216 (vs), 1047 (w), 759 (vs), 699 (m). ^1^H-NMR (400 MHz, CDCl_3_) δ: 3.32 (d, *J* = 7.4 Hz, 4H, NCH), 3.19 (d, *J* = 7.2 Hz, 4H, NCH), 1.89–1.48 (m, 22H, H_Cy_), 1.31–0.95 (m, 18H, H_Cy_), 0.92–0.78 (m, 4H, H_Cy_). ^13^C-NMR (75 MHz, CDCl_3_) δ: 162.6 (CO), 53.4 (CH_2_), 37.3 (CH), 36.0 (CH), 30.1, 30.7, 26.5, 26.3, 26.0, 25.9; HRMS (ESI) calcd. for C_30_H_53_N_4_O_2_ [M + H]^+^ 501.4169, found 501.4170.

### 3.3. Synthesis of Rotaxanes

#### 3.3.1. General Procedure for the Preparation of [2]Rotaxanes **3** and [2H]-**3**

The thread (1 mmol), **2** or [2H]-**2**, and Et_3_N (3.4 mL, 24 mmol) in CHCl_3_ (250 mL) were stirred whilst solutions of *p*-xylylenediamine (1.63 g, 12 mmol) in CHCl_3_ (40 mL) and 3,5-pyridinedicarbonyl dichloride (2.45 g, 12 mmol) in CHCl_3_ (40 mL) were simultaneously added over a period of 3.5 h using a motor-driven syringe pump. Afterwards the resulting suspension was filtered through a Celite pad and the solvent removed under reduced pressure. The resulting solid was subjected to column chromatography (silica gel, CHCl_3_:MeOH, 98:2) to yield unconsumed thread, [2]rotaxane and [2]catenane.

*[2]rotaxane [2H]-***3**. 4% yield. White solid. m.p. > 300 °C. IR (Nujol): 3579 (w), 3354 (w), 3019 (s), 2921 (w), 1658 (m), 1527 (m), 1429 (w), 1215 (vs), 1045 (w), 758 (vs), 699 (s). ^1^H-NMR (400 MHz, CDCl_3_) δ: 9.48 (s, 4H, H_B_), 8.95 (s, 2H, H_C_), 8.28 (s, 4H, NH_D_), 7.26 (s, 8H, H_F_), 5.53 (s, 2H, NH_a_), 4.56 (s, 8H, H_E_), 2.76 (d, *J* = 5.7 Hz, 8H, H_b_), 1.67-1.54 (m, 12H, H_Cy_), 1.46–1.31 (m, 12H, H_Cy_), 1.04–0.93 (m, 12H, H_Cy_), 0.70–0.57 (m, 4H, H_Cy_). ^13^C-NMR (100 MHz, CD_2_Cl_2_) δ: 165.2 (CO_thread_), 157.8 (CO_macrocycle_), 152.6, 137.8 (C), 131.1, 129.4, 129.1 (C), 55.1 (CH_2_), 45.5 (CH_2_), 37.1 (CH), 31.4, 26.8, 26.3. HRMS (ESI) calcd. for C_60_H_81_N_10_O_6_ [M + H]^+^ 1037.6341, found 1037.6344.

*[2]rotaxane* **3**. 10% yield. Orange solid. m.p. > 220 °C (decomp.). IR (Nujol): 3385 (m), 3022 (m), 2933 (s), 2850 (m), 2118 (m), 1670 (s), 1545 (m), 1445 (m), 1296 (m), 1224 (s), 1145 (w), 1121 (w), 765 (vs), 672 (vs). ^1^H-NMR (400 MHz, CDCl_3_) δ: 9.47 (s, 4H, H_B_), 8.73 (s, 2H, H_C_), 7.12 (d, *J* = 6.2 Hz, 4H, NH_D_), 7.06 (s, 8H, CH_F_), 4.96 (dd, *J* = 14.2 Hz, *J* = 7.3 Hz, 4H, H_E_’), 4.06 (d, *J* = 14.2 Hz, 4H, H_E_), 3.29–3.22 (m, 8H, H_b_), 1.85–1.47 (m, 22H, H_Cy_), 1.22–1.14 (m, 8H, H_Cy_), 1.07–0.84 (m, 8H, H_Cy_), 0.71–0.59 (m, 2H, H_Cy_), 0.47–0.30 (m, 4H, H_Cy_). ^13^C-NMR (75 MHz, CDCl_3_) δ: 164.1 (CO_thread_), 159.4 (CO_macrocycle_), 153.1, 136.9 (C), 130.9, 129.2, 128.0 (C), 56.5 (CH_2_), 56.4 (CH_2_), 44.4 (CH_2_), 37.8 (CH), 37.3 (CH), 31.8, 30.9, 26.0, 25.6. HRMS (ESI) calcd. for C_60_H_79_N_10_O_6_ [M + H]^+^ 1035.6184, found 1035.6189.

### 3.4. Chemical Exchange of [2]Rotaxanes ***3*** and [2H]-***3***

#### 3.4.1. Reduction Protocol

To a solution of the [2]rotaxane **3** (55 mg, 0.05 mmol) in chloroform (5 mL) was added hydrazine monohydrate (5 μL) in one go. The orange solution was transformed to a colorless solution in less than 5–10 min. The reaction mixture was dried with a high vacuum pump to afford the [2]rotaxane [2H]-**3** (54 mg, 0.05 mmol) as a colorless solid.

#### 3.4.2. Oxidation Protocol

To a solution of the [2]rotaxane [2H]-**3** (39 mg, 0.04 mmol) in dichloromethane (5 mL) were added pyridine (4 μL) and *N*-bromosuccinimide (7 mg, 0.04 mmol). The resulting orange solution was stirred at 25 °C for 30 min. Then the reaction mixture was diluted with dichloromethane (10 mL) and sequentially washed with water (25 mL), 5% aqueous solution of Na_2_S_2_O_3_ (20 mL) and saturated solution of NaHCO_3_ (2 × 20 mL). The organic phase was dried with MgSO_4_, and concentrated under reduced pressure to afford the [2]rotaxane **3** (37 mg, 0.04 mmol) as an orange solid.

### 3.5. Synthesis of Rotaxane ***4***

To a solution of the [2]rotaxane **3** (15 mg, 0.014 mmol) in chloroform (0.5 mL) was added *m*-CPBA (7.5 mg, 3 equiv.). The resulting solution was stirred at 25 °C for 2 h. Then the reaction mixture was diluted with chloroform (5 mL) and sequentially washed with a saturated solution of NaHCO_3_ (2 × 10 mL) and brine (10 mL). The organic phase was dried with MgSO_4_, and concentrated under reduced pressure to afford the [2]rotaxane **4** (12 mg, 81%) as an orange solid. m.p. > 300 °C. ^1^H-NMR (400 MHz, CDCl_3_) δ: 8.97 (d, *J* = 1.0 Hz, 4H, H_B_), 8.30 (s, 2H, H_C_), 7.14 (d, *J* = 6.3 Hz, 4H, NH_D_), 7.05 (s, 8H, H_F_), 4.96 (dd, *J* = 14.3 Hz, *J* = 7.6 Hz, 4H, H_E_’), 4.03 (dd, *J* = 14.3 Hz, *J* = 1.8 Hz, 4H, H_E_), 3.32–3.27 (m, 8H, H_b_), 1.85–1.55 (m, 18H, H_Cy_), 1.16–0.98 (m, 20H, H_Cy_), 0.71–0.65 (m, 2H, H_Cy_), 0.54–0.43 (m, 4H, H_Cy_). ^13^C-NMR (100 MHz, CDCl_3_) δ: 161.6 (CO_thread_), 159.4 (CO_macrocycle_), 142.3, 136.6 (C), 132.4, 129.2, 119.2 (C), 56.5 (CH_2_), 56.3 (CH_2_), 44.5 (CH_2_), 37.7 (CH), 37.3 (CH), 31.8, 31.1, 26.0, 25.9, 25.4. HRMS (ESI) calcd. for C_60_H_79_N_10_O_8_ [M + H]^+^ 1067.6077, found 1067.6045.

### 3.6. Synthesis of Rotaxane ***5***

To a solution of the [2]rotaxane **3** (10 mg, 0.01 mmol) in chloroform (0.5 mL) was added trifluoroacetic acid (2.2 μL, 3 equiv.). The resulting solution was stirred at room temperature for 1 h. Then the solvent and excess of TFA were removed under vacuum, affording the [2]rotaxane **5** (11 mg, 93%) as an brown solid. m.p. > 300 °C. ^1^H-NMR (400 MHz, CDCl_3_) δ: 9.47 (dd, *J* = 1.8 Hz, 4H, H_B_), 8.78 (t, *J* = 1.8 Hz, 2H, H_C_), 7.14 (d, *J* = 5.8 Hz, 4H, NH_D_), 7.06 (s, 8H, CH_F_), 4.96 (dd, *J* = 14.3 Hz, *J* = 7.4 Hz, 4H, H_E_’), 4.08 (dd, *J* = 14.2 Hz, *J* = 2.0 Hz, 4H, H_E_), 3.29-3.24 (m, 8H, H_b_), 1.85-1.47 (m, 18H, H_Cy_), 1.42–0.88 (m, 20H, H_Cy_), 0.71–0.59 (m, 2H, H_Cy_), 0.47–0.32 (m, 4H, H_Cy_). ^19^F-NMR (282 MHz, CDCl_3_) δ: 75.87.

### 3.7. Synthesis of Rotaxane ***6***

To a solution of the [2]rotaxane **3** (15 mg, 0.014 mmol) in acetonitrile (0.5 mL) was added allyl iodide (7.3 mg, 3 equiv.). The resulting solution was stirred at 70 °C for 48 h. Then the solvent was removed under vacuum. The resulting residue was washed with Et_2_O and CHCl_3_ to afford the [2]rotaxane **6** (14 mg, 73%) as an brown solid. m.p. > 300 °C. ^1^H-NMR (300 MHz, DMSO-*d*_6_) δ: 9.67 (d, *J* = 1.3 Hz, 4H, H_B_), 89.39 (s, 2H, H_C_), 8.87 (t, *J* = 5.2 Hz, 4H, NH_D_), 7.04 (s, 8H, H_F_), 6.24–6.11 (m, 2H, H_H_), 5.66–5.42 (m, 4H, H_G+I_), 4.45 (s, 8H, CH_E_), 3.23 (d, *J* = 6.8 Hz, 4H, CH_b_), 3.07 (d, *J* = 7.2 Hz, 4H, CH_b’_), 1.80–0.88 (m, 38H, H_Cy_), 0.71–0.62 (m, 2H, H_Cy_), 0.20–0.05 (m, 4H, H_Cy_). ^13^C-NMR (75 MHz, DMSO-*d*_6_) δ: 160.7 (CO_thread_), 158.5 (CO_macrocycle_), 146.2, 141.4 (C), 136.2, 134.3, 131.1 (C), 128.0, 123.1 (CH_2_), 63.2 (CH_2_), 54.4 (CH_2_), 54.2 (CH_2_), 43.6 (CH_2_), 36.0 (CH_2_), 30.5 (CH), 29.7 (CH), 25.9, 25.8, 25.7, 25.3, 24.9. HRMS (ESI) calcd. for C_66_H_87_N_10_O_6_ [M + H]^+^ 1115.6805, found 1115.6777.

## 4. Conclusions

In summary, we have described the assembly of two hydrogen-bonded [2]rotaxanes having a nitrogenated binding site and two pyridine rings in the macrocycle. A close inspection of their ^1^H NMR spectra reveals a deep dissimilarity in the dynamic behavior of the cyclic component as consequence of the distinguishable affinity of the binding sites for the macrocycle and the non-covalent interactions between both interlocked components. The chemical interconversion between the azo and hydrazo forms of the [2]-rotaxane facilitated the development of a chemically-switchable interlocked system enable to exchange two different dynamic states as a function of the oxidation level of the binding site of one of the entwined components. Moreover, we functionalized the pyridine-based macrocycle by oxidation, protonation and alkylation reactions, finding a new chemical means of exchange the dynamics of such interlocked systems.

## Figures and Tables

**Table 1:** Kinetic and thermodynamic parameters for macrocycle pirouetting obtained from variable-temperature ^1^H NMR spectra of the [2]rotaxanes in deuterated solvents. Δν is the separation, in Hz, between the resonance peaks of axial (H_Eax_) and equatorial (H_E’eq_) benzylic hydrogens at low temperature, k_c_ is the rate exchange in s^−1^ and T_c_ is the coalescence temperature in K. ΔG^≠^ is the energy barrier (free energy of activation) of the ring rotation process (see Supplementary Materials).

Entry	Rotaxane	δ (H_Eeq_); δ (H_E’ax_) ppm	Δν/Hz	k_c_/s^−1^	T_c_^1^/K	ΔG^≠ 2^/kcal mol^−1^
1	[2H]-**3**	4.64; 4.33	128	290	228	10.7
2	**3**	4.96; 4.06	360	802	413	19.1
3	**4**	4.98; 4.00	392	873	408	18.6
4	**5**	4.96; 4.07	356	793	413	19.0
5	[2H]-**7** ^3^	4.78; 4.22	237	527	228	10.4
6	**7**^3^	5.14; 3.67	560	1245	333	14.8

^1^ NMR temperature calibration was performed using pure methanol (low temperature) or ethylene glycol (high temperature) samples; ^2^ Value ± 0.2; ^3^ Values reported in ref. [27].

## Supplementary Materials

Supplementary materials can be accessed online. ^1^H- and ^13^C-spectral data and variable-temperature NMR experiments.
