# Supplementary material for: Effects on Rotational Dynamics of Azo and Hydrazodicarboxamide-Based Rotaxanes
Source: Molecules. 2017 Jun 28;22(7):1078. doi: 10.3390/molecules22071078 (PMC6152038; doi:10.3390/molecules22071078)

<u>Supporting information</u>

Effects on Rotational Dynamics of Azo and Hidrazodicarboxamide-based Rotaxanes

Adrian Saura-Sanmartin, Juan S. Martinez-Espin, Alberto Martinez-Cuezva,* Mateo Alajarin, Jose Berna*

Departamento de Química Orgánica, Facultad de Química, Regional Campus of International Excellence "Campus Mare Nostrum", Universidad de Murcia, E-30100 Murcia (Spain).

Email: ppberna@um.es; amcuezva@um.es

Table of

Contents………………………………………………………………………………………… S2

1. Variable-Temperature NMR Experiments ……………………………………………………….. S3

2. ^{1}H and ^{13}C NMR spectra of synthesized compounds ……………………………………………… S6

1. Variable-Temperature NMR Experiments

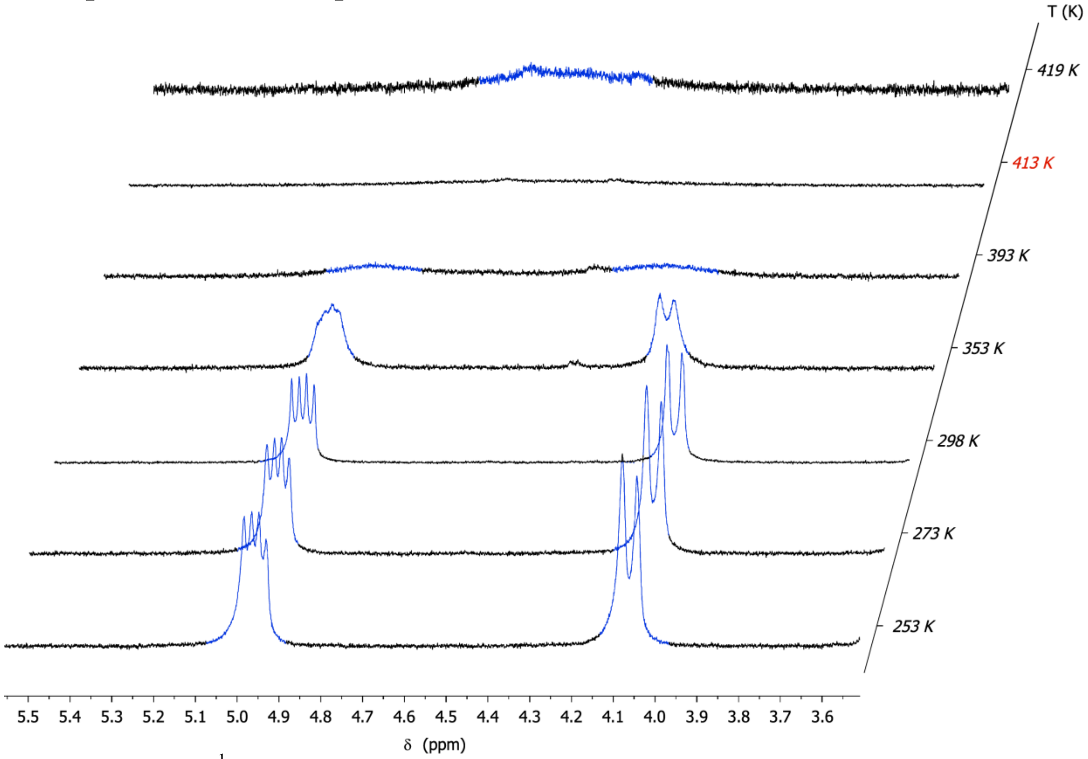

Figure S1. Variable temperature ^{1}H NMR spectra (stacked expansions of the aliphatic region, 400 MHz) of rotaxane **3**.

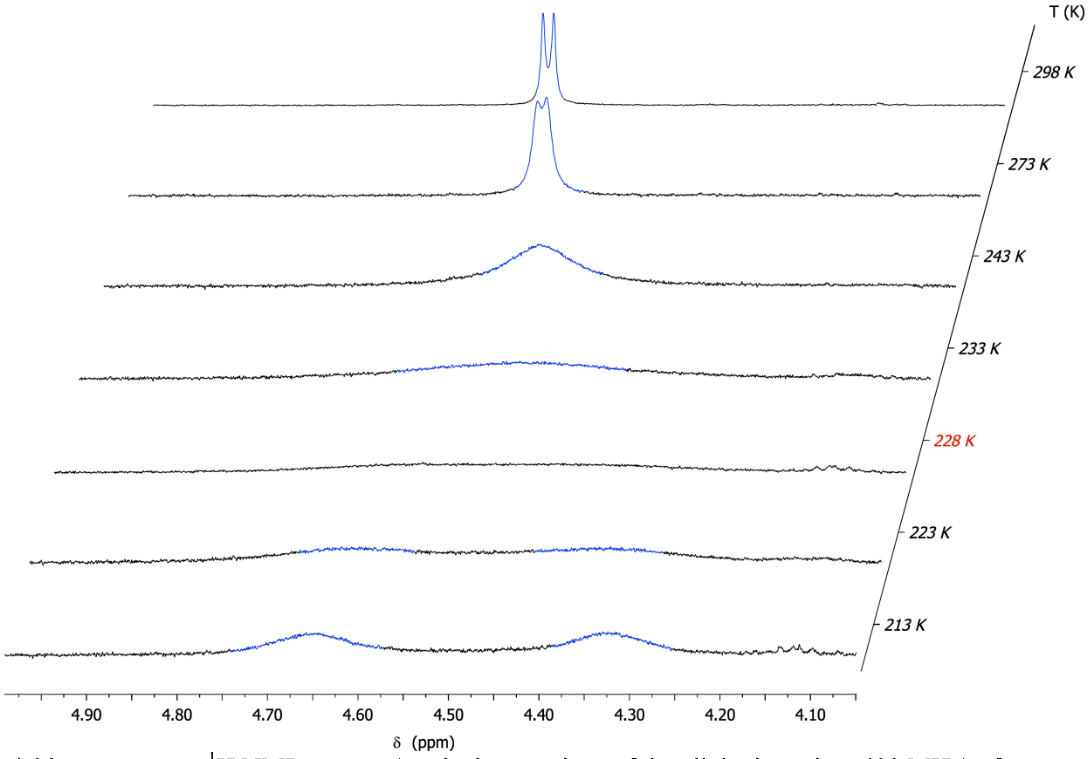

Figure S2. Variable temperature ^{1}H NMR spectra (stacked expansions of the aliphatic region, 400 MHz) of rotaxane [2H]-**3**.

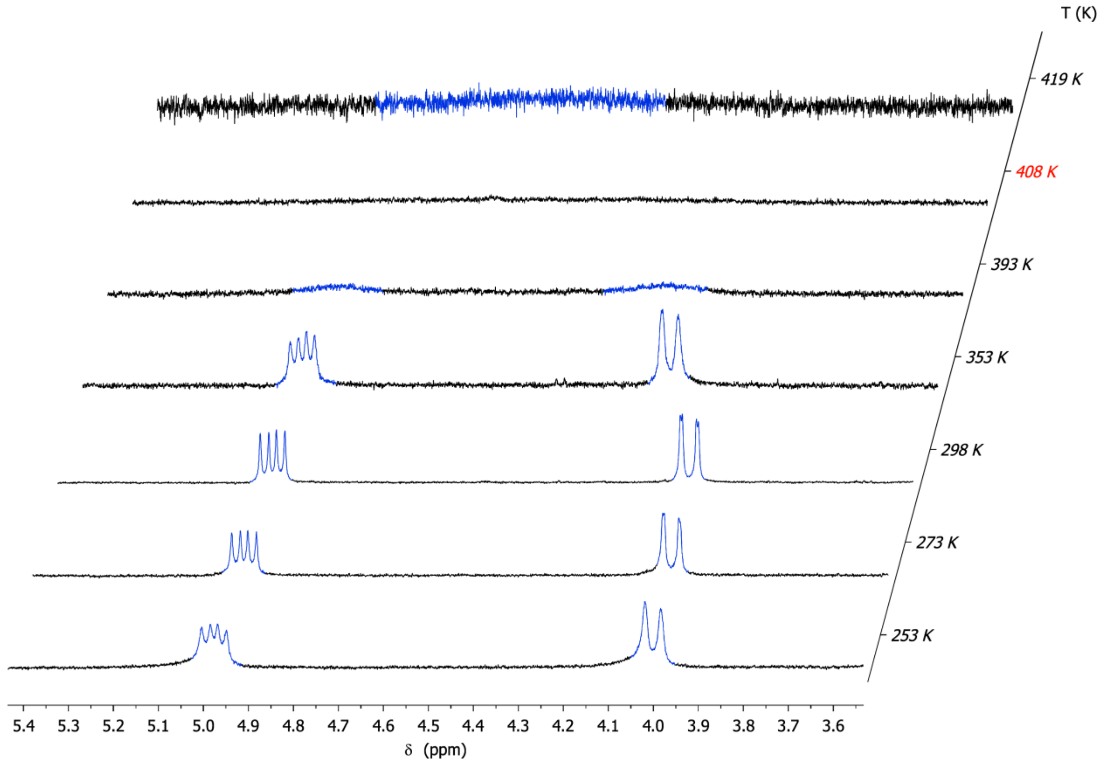

Figure S3. Variable temperature ^{1}H NMR spectra (stacked expansions of the aliphatic region, 400 MHz) of *N*-oxide-based rotaxane **4**.

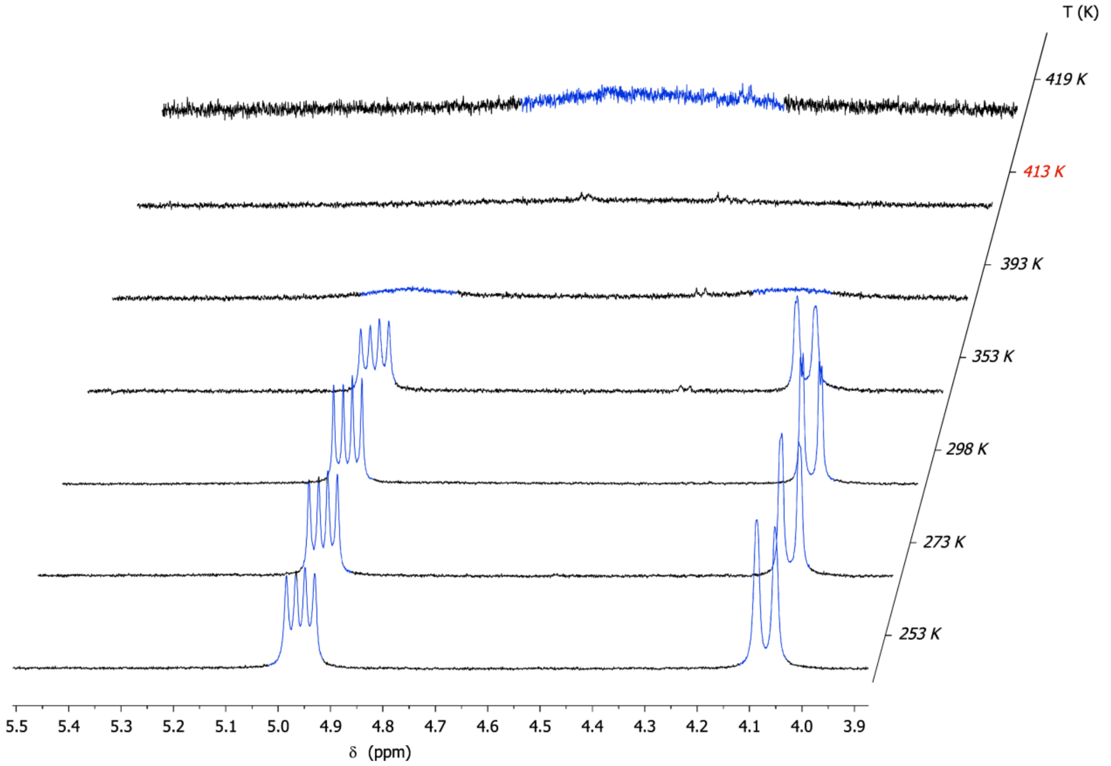

Figure S4. Variable temperature ^{1}H NMR spectra (stacked expansions of the aliphatic region, 400 MHz) of salt **5**.

Free energies of activation were calculated using the Eyring equation, $\Delta G_c^{\neq} = -RT_c \cdot \ln(k_c h/k_b T_c)$, where $k_c = (\pi\Delta\upsilon)/\sqrt{2}$ or $kc = \pi\sqrt{(\Delta\upsilon^2 + 6J^2)}/\sqrt{2}$ and *R*, *h* and *kb* are the gas, Planck and Boltzmann constants, respectively.

2. ^{1}H and ^{13}C NMR Spectra of synthesized compounds

[2H]-**2** (^{1}H NMR, 400 MHz, $CDCl_3$, 298K)

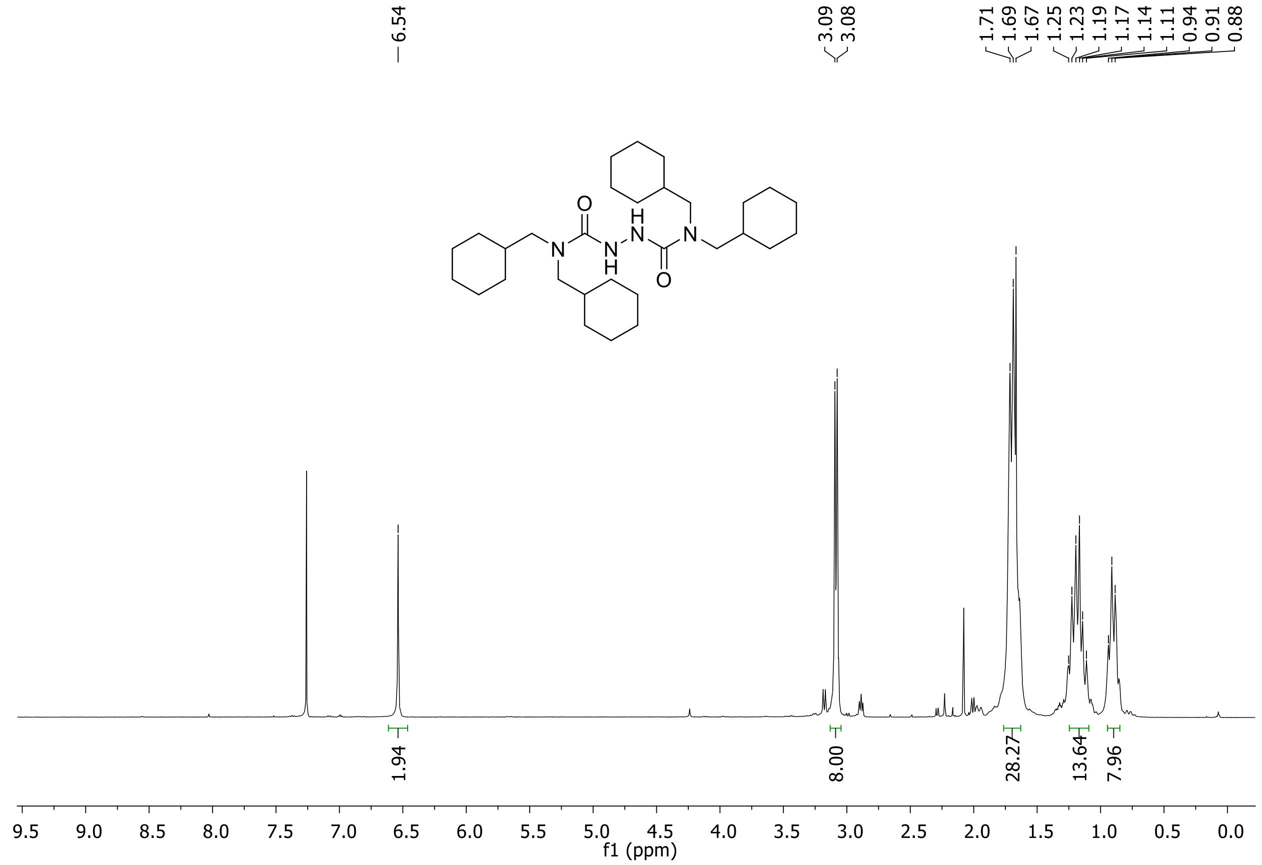

[2H]-**2** (^{13}C NMR, 100 MHz, $CDCl_3$, 298K)

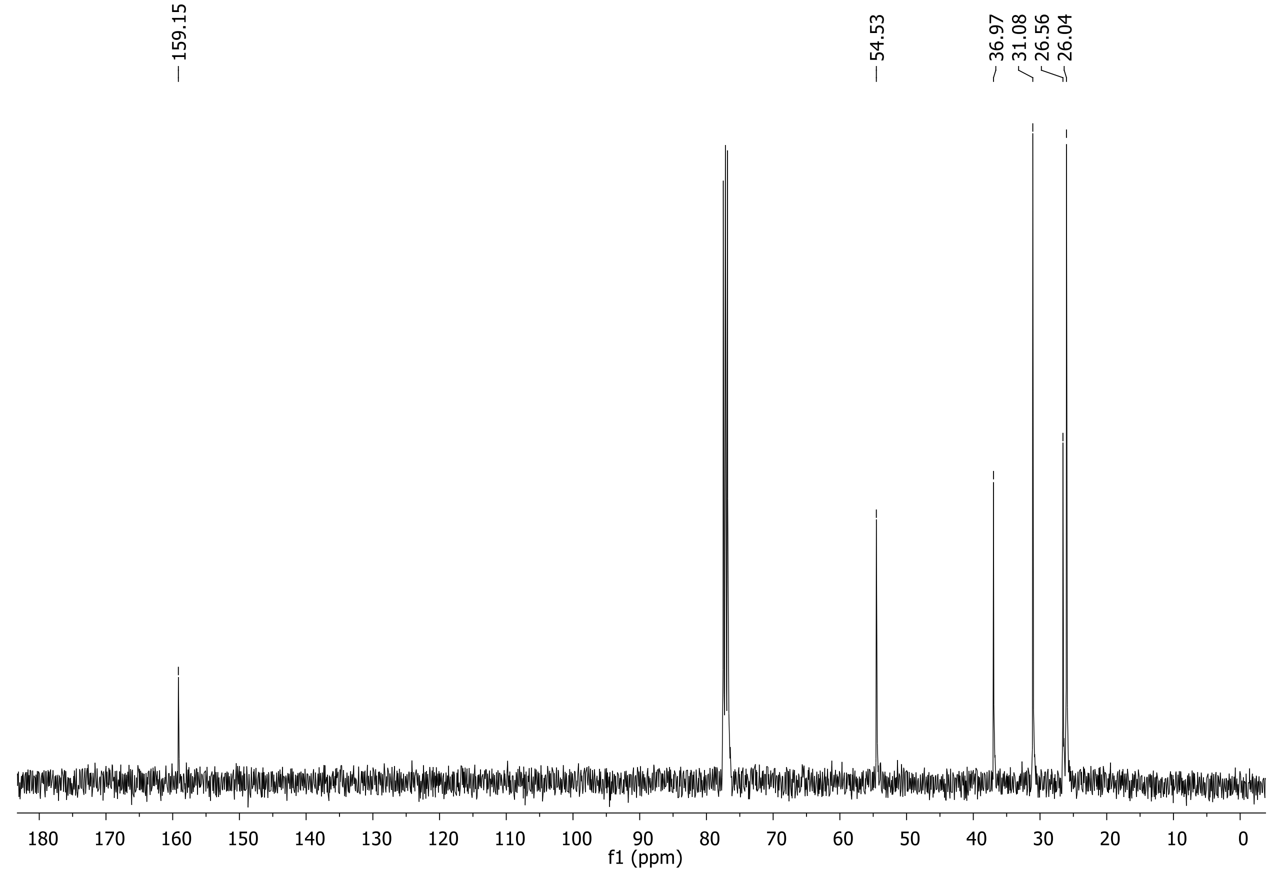

2 (^{1}H NMR, 400 MHz, $CDCl_3$, 298K)

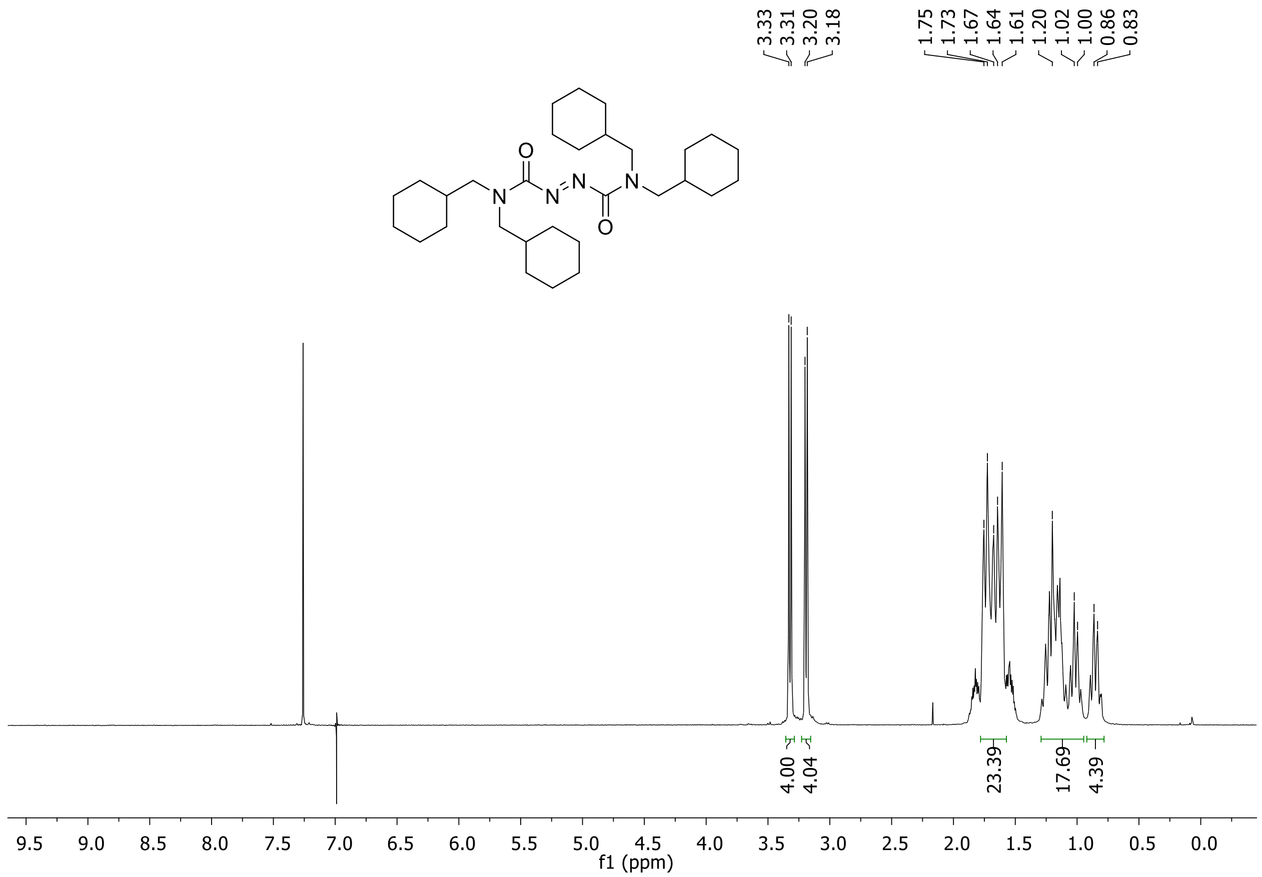

2 (^{13}C NMR, 100 MHz, $CDCl_3$, 298K)

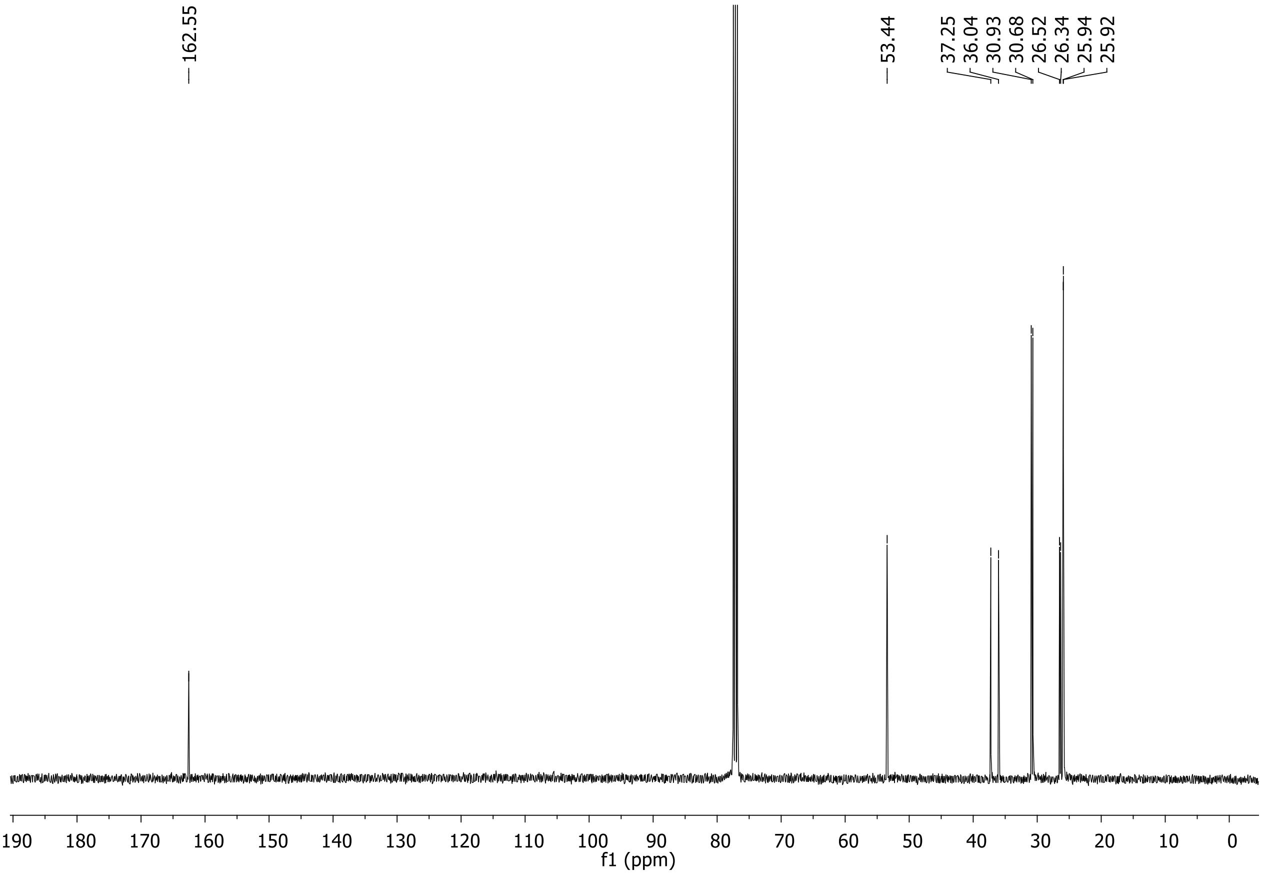

[2H]-**3** (^{1}H NMR, 400 MHz, $CDCl_3$, 298K)

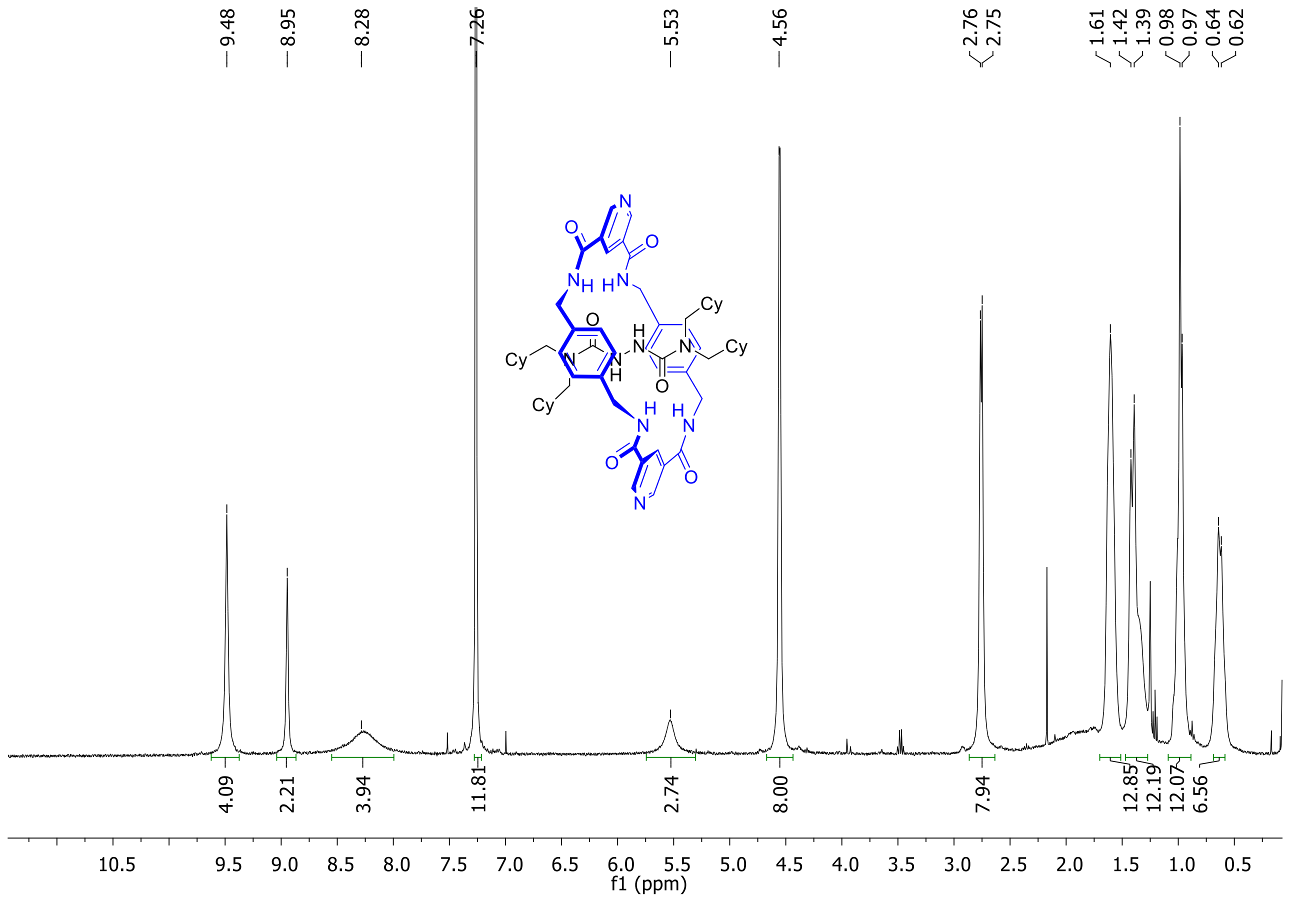

[2H]-**3** (^{13}C NMR, 100 MHz, CD_2Cl_2, 298K)

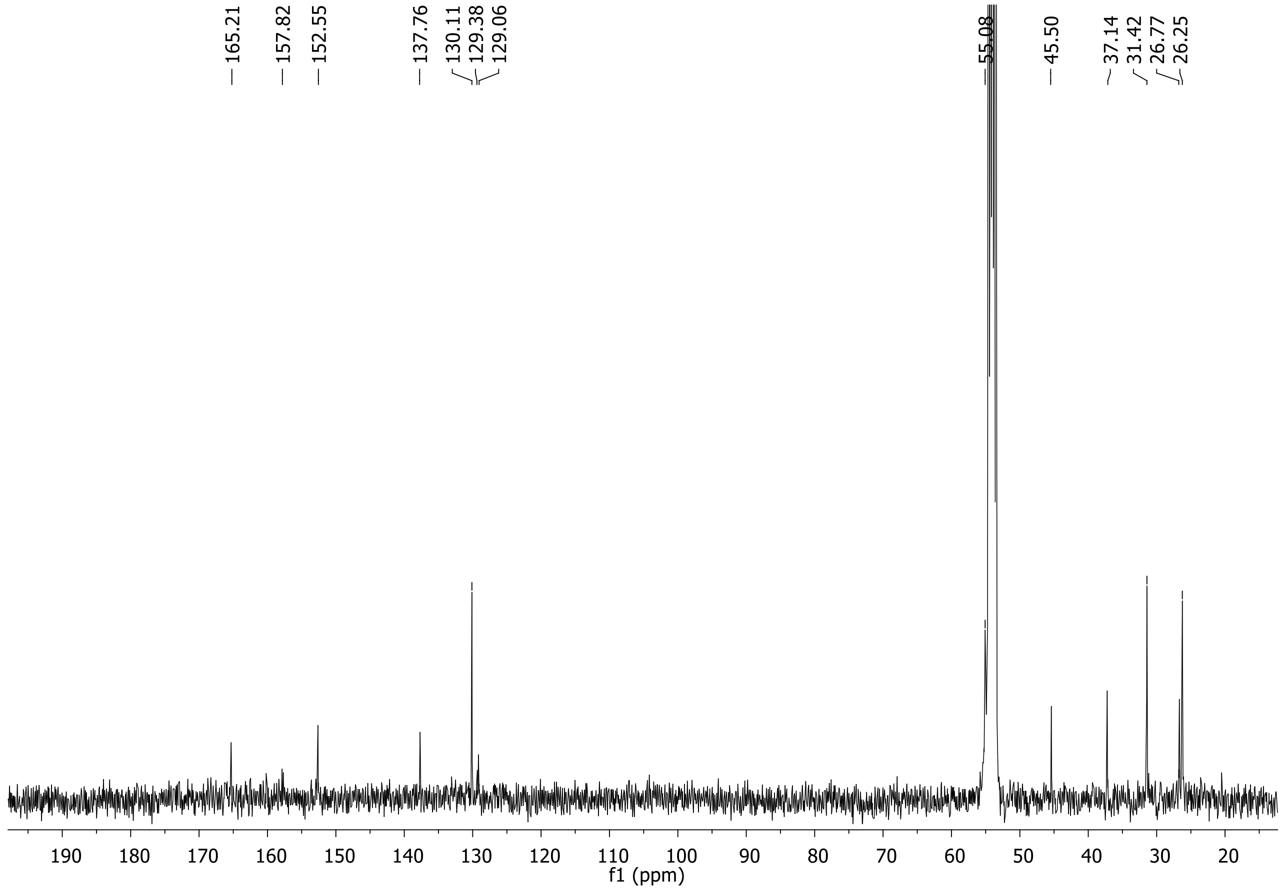

3 (^{1}H NMR, 400 MHz, $CDCl_3$, 298K)

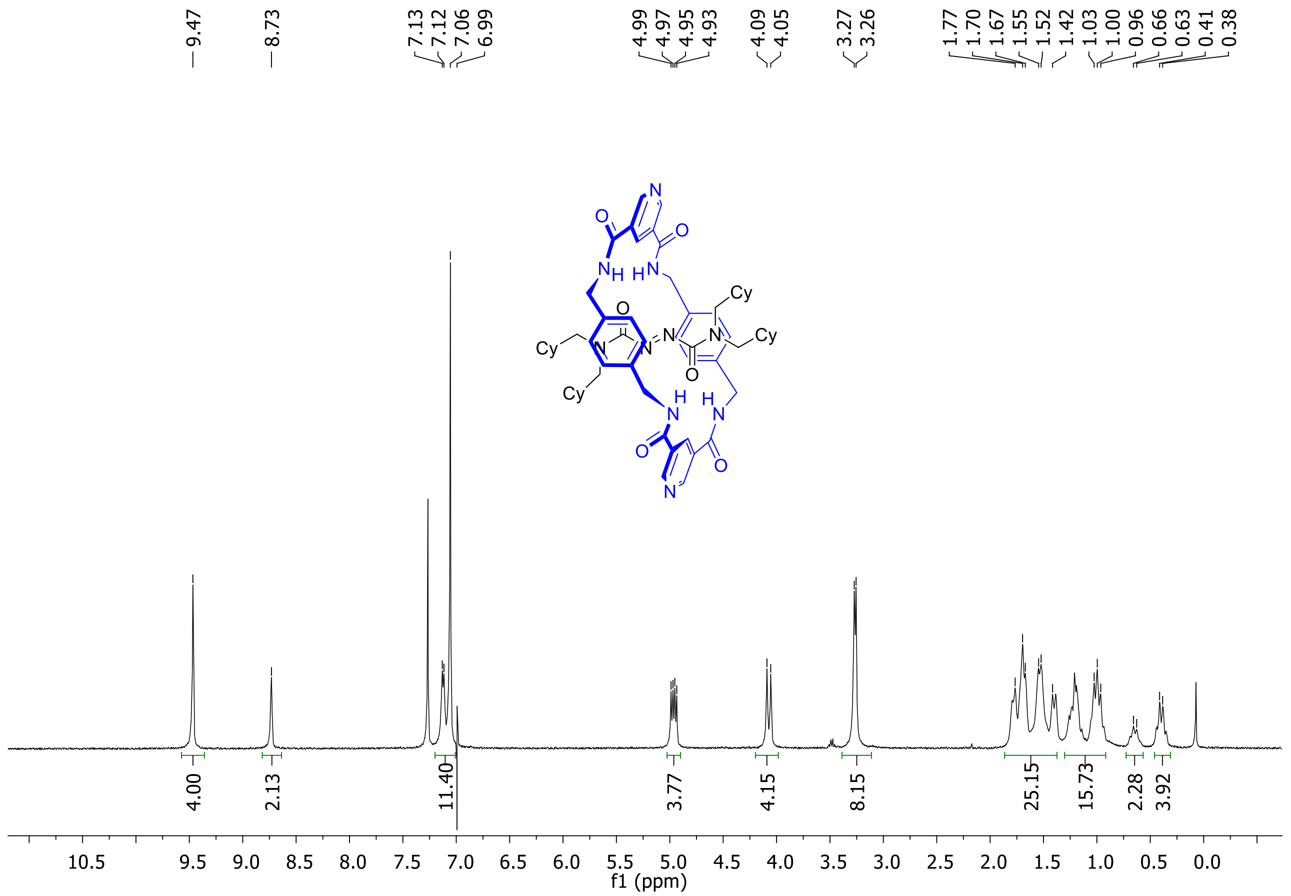

3 (^{13}C NMR, 75 MHz, $CDCl_3$, 298K)

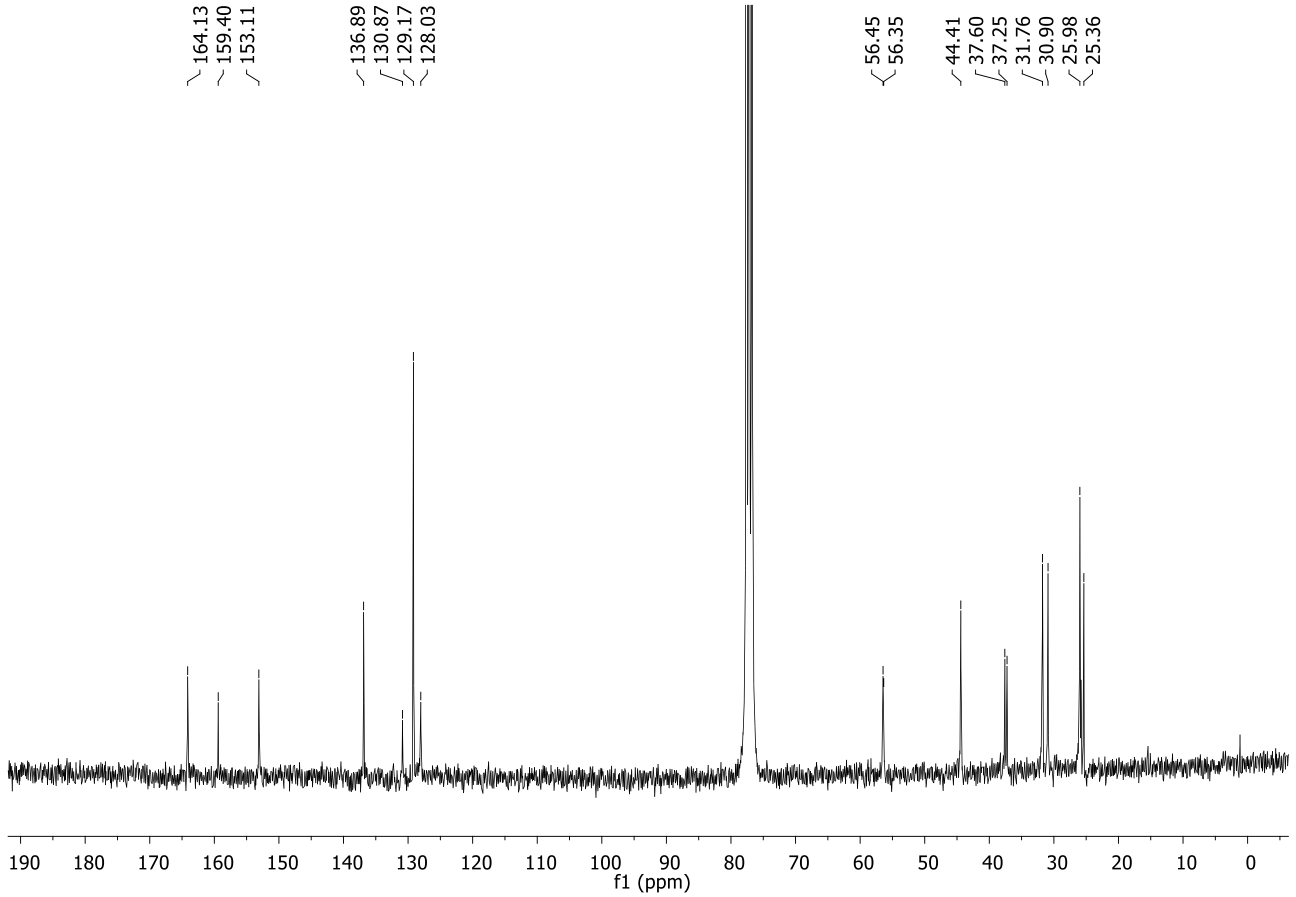

4 (^{1}H NMR, 400 MHz, $CDCl_3$, 298K)

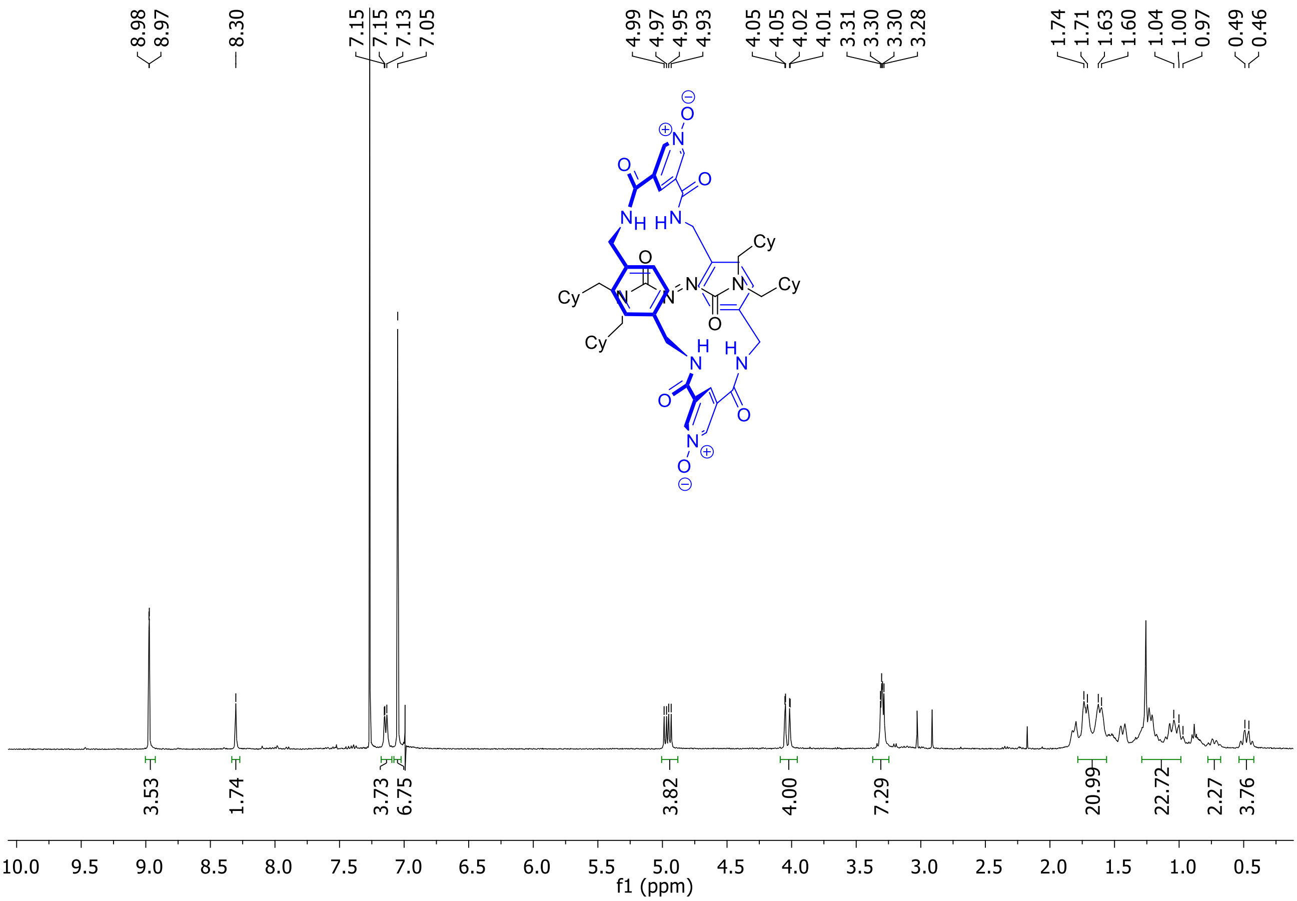

4 (^{13}C NMR, 100 MHz, $CDCl_3$, 298K)

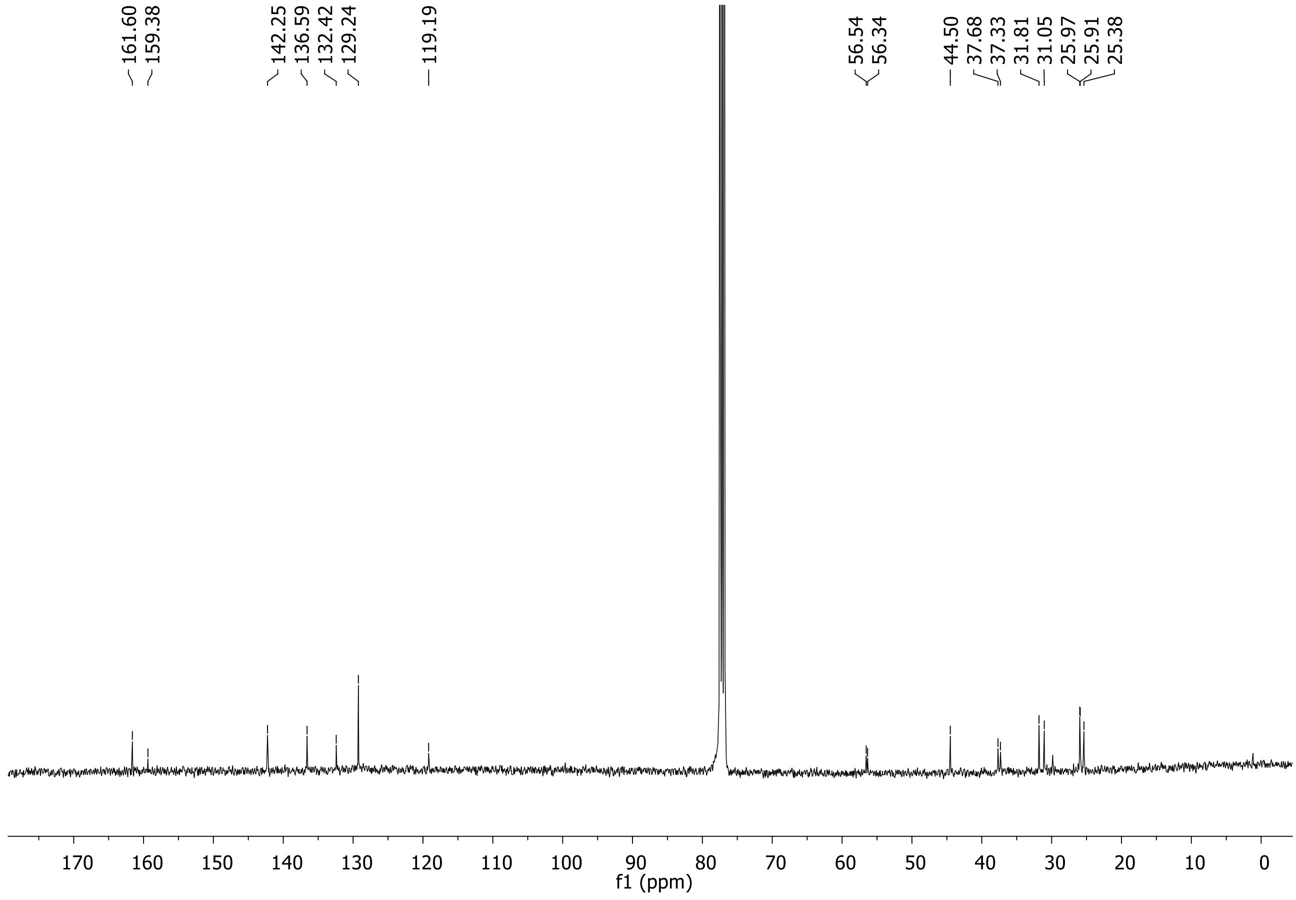

5 (^{1}H NMR, 400 MHz, $CDCl_3$, 298K)

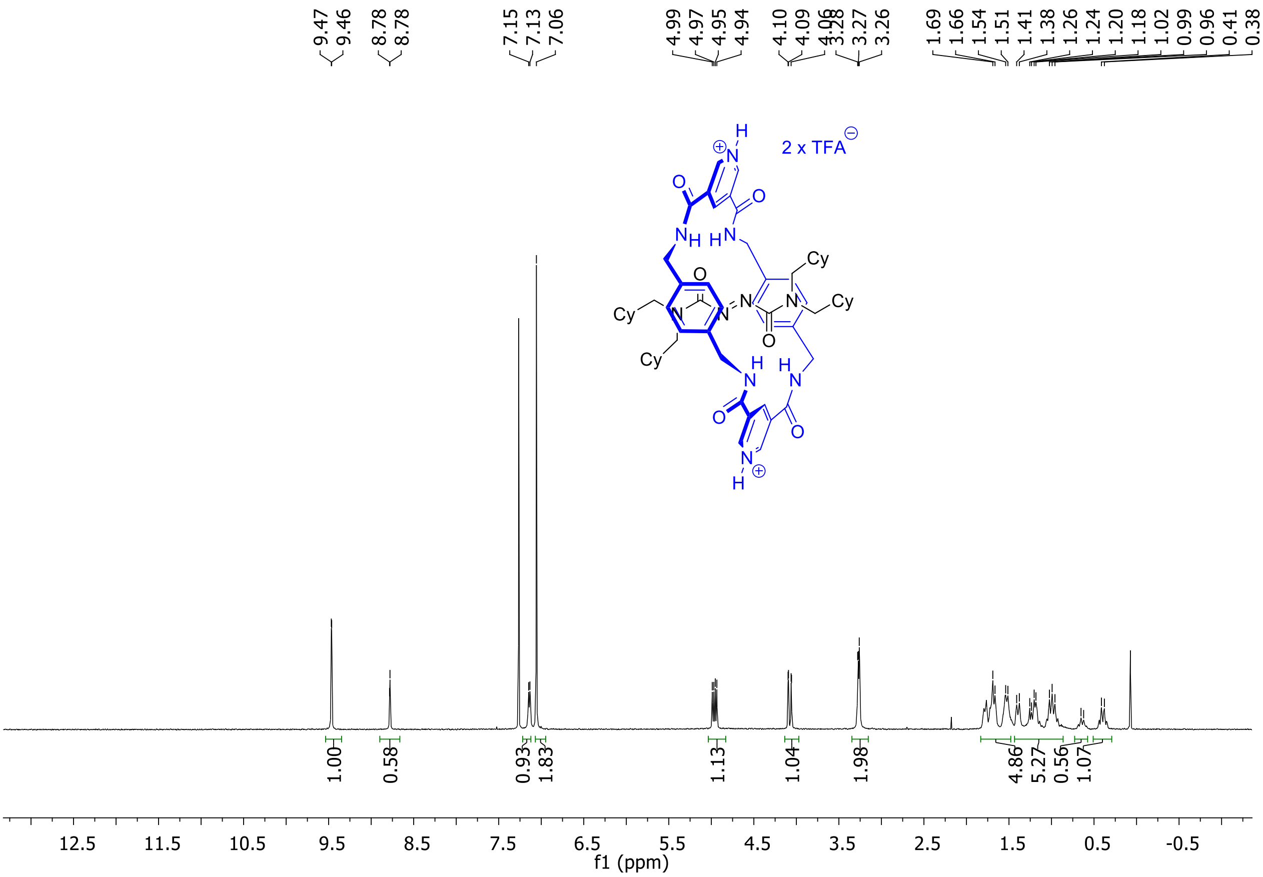

5 (^{19}F NMR, 282 MHz, CHCl3, 298K)

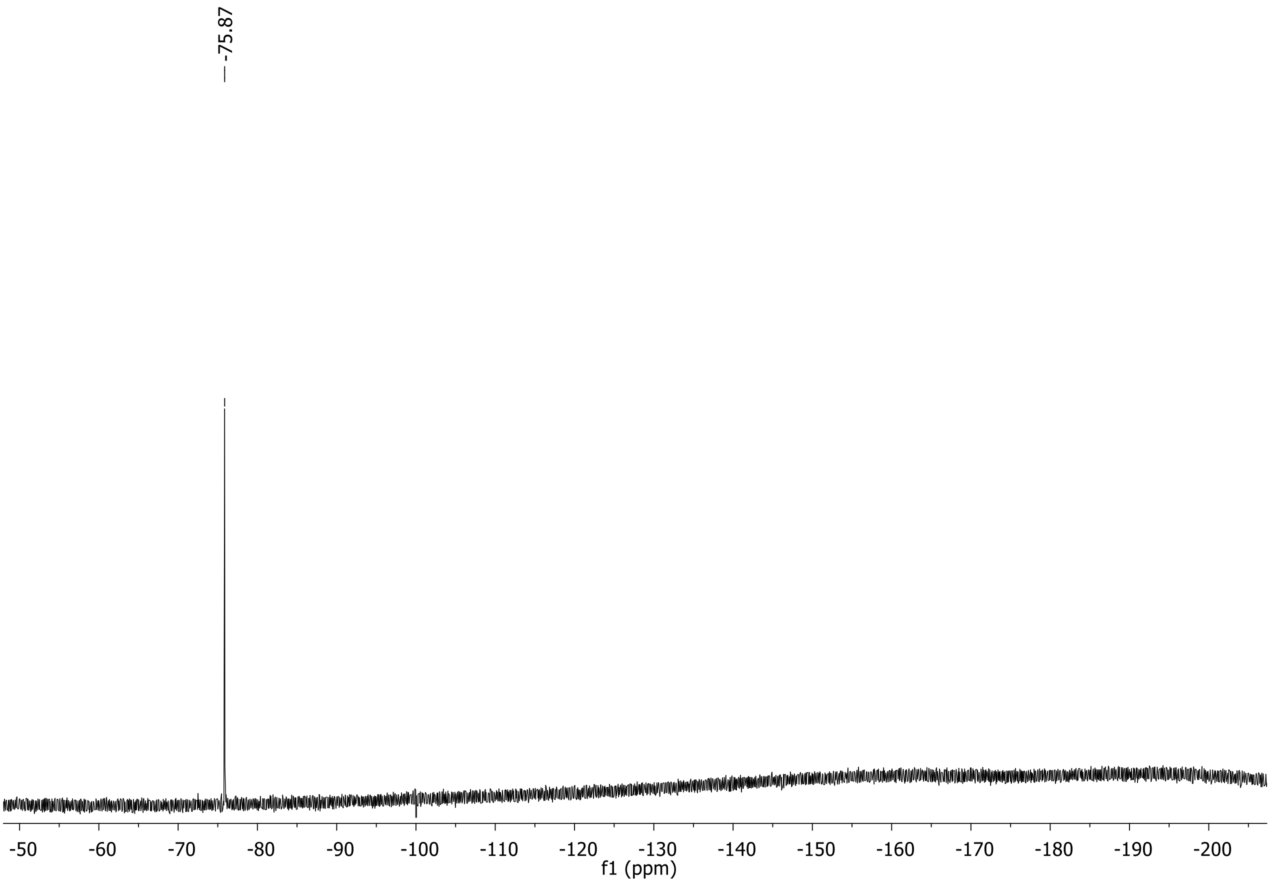

6 (^{1}H NMR, 300 MHz, DMSO-*d6*, 298K)

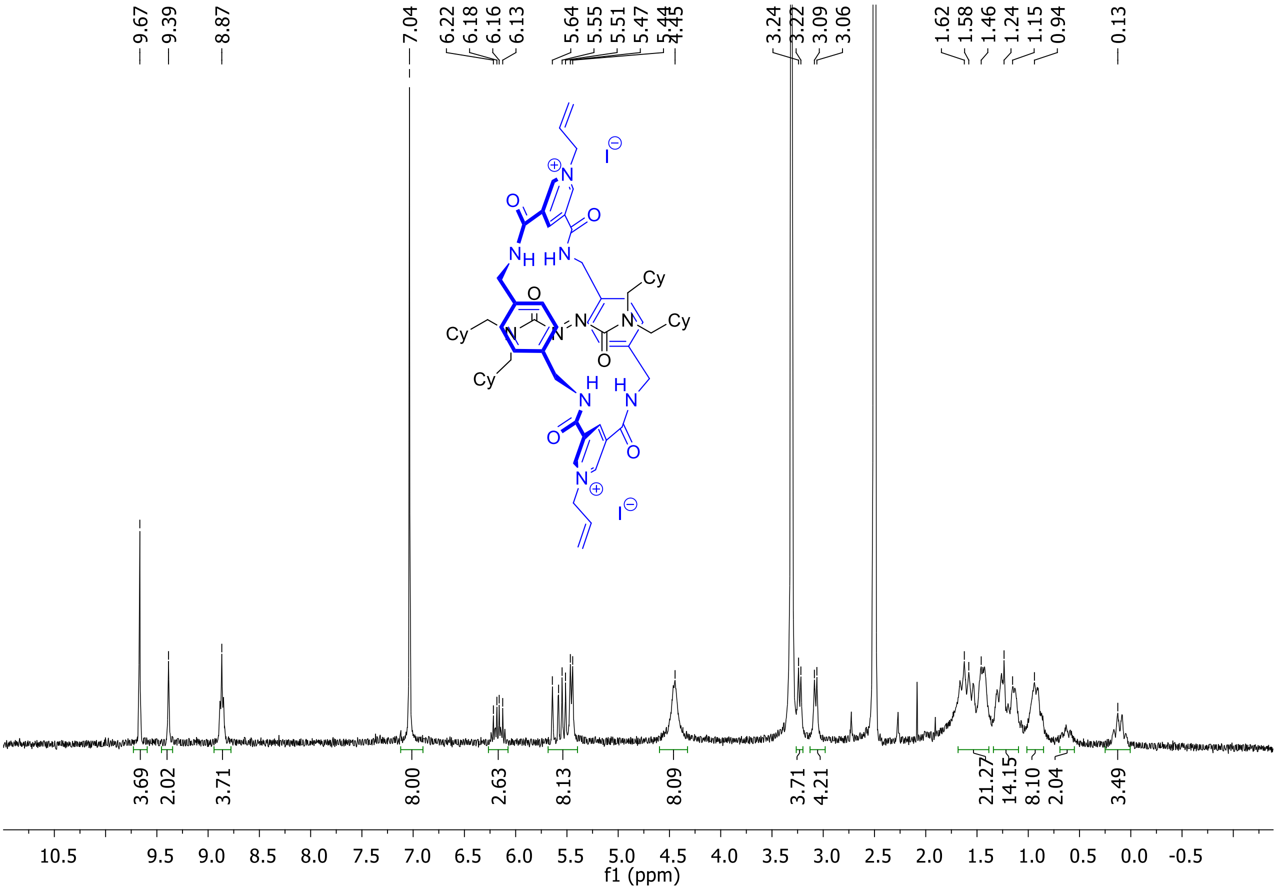

6 (^{13}C NMR, 75 MHz, DMSO-*d6*, 298K)

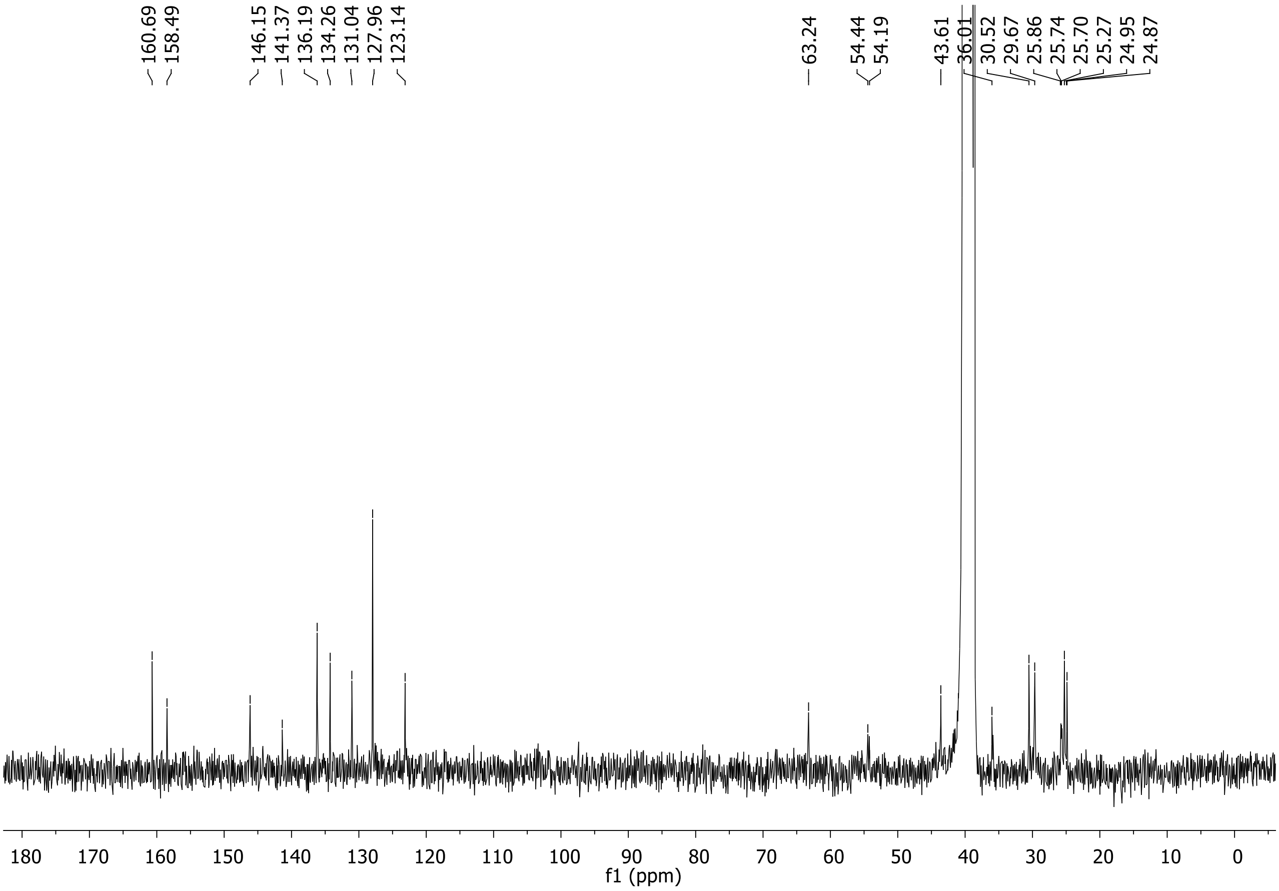

Supplement: Supplementary file 1 [file molecules-22-01078-s001.pdf]
